# A mouse model of allergic conjunctivitis with mucosal immune-related gene and circRNA dysregulation

**DOI:** 10.3389/fimmu.2026.1744124

**Published:** 2026-03-06

**Authors:** Hongyu Zhang, Yan Qi, Qing Leng, Yaning Qin, Hong Zhang, Bing Wu

**Affiliations:** 1 Department of Neurosurgery, China-Japan Union Hospital of Jilin University, Changchun, China; 2 Department of Immunology, College of Basic Medical Sciences, Jilin University, Changchun, China; 3 Department of Ophthalmology, China-Japan Union Hospital of Jilin University, Changchun, China

**Keywords:** allergic conjunctivitis, circRNA, immune infiltration, immune-related genes, mucosal immunity, transcriptomic analysis

## Abstract

Allergic conjunctivitis (AC) is an inflammatory ocular condition triggered by allergens like pollen. Although general allergic mechanisms are well characterized, the immune specificity of the ocular mucosa remains unclear. This study investigates immune-related mRNAs and circRNAs to uncover novel molecular pathways in AC pathogenesis. Using a ragweed pollen-induced murine AC model, conjunctival tissues were subjected to RNA sequencing. DESeq2-based differential expression analysis revealed dysregulated mRNAs and circRNAs, with significant enrichment in the IL-17 signaling pathway. Key IL-17-associated genes (*Tnfrsf4*, *Cxcl1*, *Lef1*) and co-expressed circRNAs (Circ9626, Circ2598, etc.) were markedly upregulated. Immune infiltration analysis confirmed a mixed Th2/Th17 response with notable neutrophil involvement. These findings highlight the potential involvement of the Th17/IL-17 axis beyond classical Th2 immunity and construct a putative circRNA–IL-17 co-expression network, providing a comprehensive transcriptomic landscape and identifying potential candidates for future therapeutic exploration in AC.

## 1 Introduction

Ragweed, belonging to the Asteraceae family, is one of the most significant allergen sources within its genus due to its highly sensitizing pollen (1). Ragweed triggers pollen-related allergic reactions in approximately 40% of atopic individuals in the United States and Europe (1, 2); in certain parts of Asia, such as Japan, the prevalence of Allergic conjunctivitis (AC) reaches as high as 30% (3). A study in Korea found that excessive diurnal temperature variation might lead to an increase in airborne allergens, including pollen, consequently raising the incidence of AC (4). Furthermore, an analysis in Beijing, China, demonstrated that increased atmospheric pollen concentrations are closely associated with a rise in AC consultations (5). To date, research on diseases induced by ragweed pollen has primarily focused on allergic rhinitis and asthma, with relatively fewer studies addressing allergic conjunctivitis as an independent entity. Therefore, this study established a mouse model of ragweed pollen-induced allergic conjunctivitis to investigate the pathological mechanisms underlying pollen-associated conjunctivitis.

Allergic conjunctivitis (AC), a prevalent ocular surface disorder, is characterized by type I hypersensitivity reactions triggered by environmental allergens such as pollen, animal dander, and dust mites. With a global prevalence of 10%-20%, AC manifests with hallmark symptoms including ocular redness, itching, burning sensation, and excessive tearing (6–9). Despite its significant impact on patients’ quality of life, AC is often overlooked in clinical practice and merely categorized as a non-infectious subtype of conjunctivitis (10, 11).

The pathogenesis of AC has been traditionally attributed to the dysregulation of Th2-mediated immune responses. Upon allergen exposure, antigen-presenting cells process and present allergens to Th2 cells, which subsequently secrete cytokines that promote IgE production by B cells. This cascade leads to mast cell activation and release of inflammatory mediators, particularly histamine, resulting in characteristic allergic symptoms (12, 13). While this classical mechanism has been extensively studied, the unique immunoregulatory mechanisms specific to the ocular surface remain poorly understood.

The ocular surface, as a direct interface with the external environment, maintains a delicate balance between immune defense and tolerance (14). Excessive inflammatory responses can compromise the ocular surface barrier, potentially leading to severe complications such as corneal ulceration and scarring (15, 16). To preserve ocular homeostasis, the conjunctival mucosa employs multiple immunoregulatory mechanisms. Goblet cells secrete immunosuppressive factors like TGF-β, while epithelial cells express immune checkpoint molecules such as PD-L1 and produce anti-inflammatory cytokines including IL-10 to create an immunotolerant microenvironment (17–19). These mechanisms differ from the immune privilege of intraocular structures, which relies on specialized barriers like the blood-ocular barrier and immunosuppressive factors present in the aqueous humor (20).

The ocular surface, despite its relatively low immune reactivity, maintains robust antimicrobial defenses to counteract the potential risk of infection. This delicate balance is achieved through specialized ocular mucosal immune regulatory mechanisms. For instance, epithelial cells express pattern recognition receptors, such as TLRs and NLRs, which detect pathogen-associated molecular patterns and initiate innate immune responses (17, 21–23). Furthermore, the secretion of antimicrobial components, including lysozyme, lactoferrin, and secretory IgA, provides immediate protection against microbial invasion while minimizing inflammatory tissue damage. These features underscore the tissue-specific nature of mucosal immune responses at the ocular surface, which are finely regulated to maintain ocular homeostasis. Recent evidence suggests that the pathogenesis of AC in the conjunctiva may differ from traditional models (17). For example, studies in ragweed-induced murine AC models have demonstrated that mast cell activation may not be the primary driver of disease (24). This finding emphasizes the need for more in-depth investigations into conjunctiva-specific immune mechanisms in AC, which could lead to the development of targeted therapeutic strategies that better address the unique immunological characteristics of the ocular surface.

Circular RNAs (circRNAs), a class of covalently closed non-coding RNAs, have emerged as key regulators in numerous biological processes (25, 26). Accumulating evidence suggests that these non-coding RNAs play pivotal roles in various allergic diseases by modulating signal transduction pathways, immune cell activity, and host-pathogen interactions (27). However, the involvement of circRNAs in AC remains largely unexplored. In this study, we established a murine AC model and utilized transcriptomic sequencing to systematically analyze mucosal-associated genes and circRNAs. By integrating bioinformatics approaches, we aimed to uncover novel insights into the immunoregulatory mechanisms underlying AC, which could contribute to the development of targeted therapeutic strategies.

## 2 Materials and methods

### 2.1 Establishment of AC model

All experimental procedures were approved by the Animal Ethics Committee of Jilin University (Approval No. 197) and strictly complied with China’s national standard GB/T 35892–2018 “Guidelines for Ethical Review of Laboratory Animal Welfare”. The study was conducted at the Laboratory Animal Center of Basic Medical Sciences, Jilin University, which holds the institutional animal care license (SYXK (Ji) 2023–0010) for SPF-grade rodents under barrier conditions.

The AC model was induced following an established protocol (28). Female BALB/c mice (SPF grade, aged 8–10 weeks; Yisi Laboratory Animal Technology Co., Ltd., Changchun, China) were maintained under controlled conditions: 12-hour light/dark cycle, ambient temperature of 22 ± 2 °C, 55 ± 5% humidity, with ad libitum access to food and water. Female BALB/c mice were chosen based on its propensity for a robust Th2-polarized immune response, pertinent to allergic disease modeling. Prior to the experiment, mice were randomly assigned to the Control group and the AC model group. Initial sensitization was performed on day 0 by footpad injection. Each mouse footpad received 100 µg of short ragweed (SRW) pollen (Greer Lab, Lenoir, NC, USA) adsorbed onto 2 mg of Imject Alum adjuvant (InvivoGen, San Diego, CA, USA) in a 100 µL volume. Control animals were administered an equivalent volume of Imject Alum alone. Commencing on day 10, 10 µL of 50 mg/mL SRW pollen solution was administered to each eye daily for five consecutive days; control mice received 10 µL of phosphate-buffered solution (PBS), which served as the diluent for SRW.

### 2.2 Histologic analysis of conjunctiva

On day 14 after the SRW pollen challenge, mice (n = 3 per group) were euthanized by intraperitoneal injection of an overdose of sodium pentobarbital (150 mg/kg), and the eyes with attached eyelids and conjunctival tissues, along with the cervical lymph nodes (CLNs), were collected. For conjunctival histology, mouse eyes with eyelids attached were isolated. Hair surrounding the eyelids was trimmed using scissors and forceps. Tissues were fixed in 4% paraformaldehyde and embedded in paraffin. Paraffin sections (5 μm thick) of the eyes and conjunctiva were prepared using a rotary manual microtome (S700, RWD, China). Hematoxylin and Eosin (H&E) staining (C0105M, Beyotime, China) was performed to assess ocular tissue morphology. Toluidine Blue staining (G2543, Solarbio, China) was used to detect mast cells (MCs). Sections were examined using a microscope system (Olympus BX53, Olympus, Japan). To minimize subjective bias, image acquisition and histological evaluation were performed by investigators who were blinded to the group allocations.

### 2.3 Immune cell infiltration of AC

Prediction of immune cell abundance was performed using the online tool Immune Cell Abundance Identifier (29) (ImmuCellAI-mouse) (https://guolab.wchscu.cn/ImmuCellAI-mouse/#!/). This platform predicts the relative abundance of multiple immune cell types within mouse tissues based on RNA-Seq or microarray expression profile data.

### 2.4 Evaluation of Th cells and neutrophils lineages by flow cytometry and immunofluorescence

To isolate lymphocytes, CLNs were collected from each mouse (n = 3 per group). CLNs were cut into small pieces, then ground using the flat end of a syringe plunger, and filtered through a 70-μm cell strainer. After centrifugation, cells were resuspended in 200 μL PBS. Cell interleukin production was stimulated using a cell stimulation cocktail (eBioscience, 3105138, USA). Cells were then labeled with flow cytometry antibodies targeting CD3 and CD4 for surface markers. Fixation and membrane permeabilization were performed using a Foxp3 Transcription Factor Staining Buffer Set (eBioscience, 00-5523-00, USA). Cells were subsequently labeled with flow cytometry antibodies targeting IFN-γ, IL-4, IL-17 to label intracellular cytokines. Finally, Th cell analysis was performed using a flow cytometer (BD Biosciences, USA).

For the preparation of single-cell suspensions from conjunctival tissue, the conjunctival tissue was minced into approximately 1-mm³ pieces using sterile scissors. The minced tissue was then transferred into 2 mL of RPMI-1640 medium (Gibco, 11875093) containing 1 mg/mL Collagenase D (Roche, 11088858001) and 0.1 mg/mL DNase I (Sigma, DN25, USA) for enzymatic digestion. The prepared single-cell suspension was stained with CD11b and Ly6g flow cytometry antibody for subsequent flow cytometry analysis. For flow cytometric gating, researchers were blinded to the experimental group assignments to minimize bias.

For immunofluorescence staining, sections were incubated with fluorescently labeled primary antibodies against Ly6g. Nuclei were then counterstained with DAPI (Sigma-Aldrich, USA). Samples were subsequently examined using a fluorescence microscope (Axio Imager Z2, Zeiss, Germany). All antibodies mentioned above used are listed in Additional file 1: **Supplementary Table S1**.

### 2.5 Cytokine quantification by ELISA

Ocular surface and conjunctival sac secretions were collected using sterile swabs under aseptic conditions in a laminar flow hood from each mouse (n = 5 per group). The swabs were then immersed in a fixed volume of sterile saline and vortexed vigorously to ensure maximal elution of the secretions. Conjunctival tissues were homogenized in ice-cold RIPA lysis buffer (supplemented with protease inhibitors) and centrifuged at 12000g for 15 min at 4 °C to harvest the supernatants. To ensure data comparability, total protein concentrations in both secretion eluates and tissue supernatants were determined using a BCA Protein Assay Kit (Thermo Fisher Scientific, 23225, USA) for normalization.

The protein levels of IL-17A (homodimer) were quantified using the Mouse IL-17A (homodimer) Uncoated ELISA Kit (Invitrogen, 88-7371, USA) according to the manufacturer’s protocol. Briefly, 96-well microplates were coated with capture antibody overnight at 4 °C. After washing with PBST, the plates were blocked with ELISA/ELISPOT Diluent for 1 h at room temperature. Standards and diluted samples were then added and incubated for 2 h. Subsequently, the plates were incubated with biotinylated detection antibody followed by Avidin-HRP conjugate. The reaction was developed with TMB substrate for 20 min in the dark and terminated with 2N H_2_SO_4_ stop solution.

The optical density (OD) was measured at 450 nm using a microplate reader. Concentrations of IL-17A were calculated using a four-parameter logistic (4-PL) curve fit. For conjunctival tissues, the results were expressed as picograms per milligram of total protein (pg/mg protein). For secretion eluates, the results were expressed as picograms per milliliter (pg/ml).

### 2.6 RNA isolation and sequencing

To overcome the limited RNA yield from individual conjunctival tissues, samples from both eyes of two mice were combined to form a single RNA pool. This pooling strategy generated five biological replicates for both the control group (derived from 10 mice) and the AC model group (derived from 10 mice). Total RNA was isolated from these pooled samples using TRIzol reagent (Invitrogen, Carlsbad, CA, USA) according to the manufacturer’s protocol. RNA integrity was initially assessed by denaturing agarose gel electrophoresis. RNA concentration was quantified using a NanoDrop 2000 spectrophotometer (Thermo Fisher Scientific, Wilmington, DE, USA), and RNA integrity was further verified using an Agilent 2100 bioanalyzer (Agilent Technologies, Santa Clara, CA, USA). Each RNA sample was subsequently split into two aliquots: one designated for transcriptome sequencing and the other reserved for reverse transcription quantitative polymerase chain reaction (RT-qPCR) validation.

RNA library preparation and sequencing services were provided by Gene Denovo Biotechnology Co., Ltd. (Guangzhou, China). To ensure the inclusion of both mRNAs and non-polyadenylated circRNAs, ribosomal RNA (rRNA) was depleted from total RNA using the Ribo-off rRNA Depletion Kit (Vazyme, N406, Nanjing, China). The rRNA-depleted RNA was then utilized to generate sequencing libraries with the VAHTS Universal V6 RNA-seq Library Prep Kit for Illumina (Vazyme, NR604, Nanjing, China). This procedure involved: (1) thermal fragmentation of the rRNA-depleted RNA; (2) first-strand cDNA synthesis using random hexamer primers; (3) second-strand cDNA synthesis incorporating end repair and dA-tailing; (4) adapter ligation; (5) size selection and purification of ligation products using VAHTS DNA Selection Beads; and (6) PCR amplification of the final library. Library quality was assessed using the DNA 1000 assay Kit (Agilent Technologies, Santa Clara, CA, USA). Finally, sequencing was conducted on an Illumina Novaseq X Plus platform. Raw sequencing data have been deposited in the Genome Sequence Archive (GSA) database.

Raw sequencing reads were first processed with fastp (v0.18.0) to remove low-quality reads and adapter sequences, retaining high-quality clean reads. Clean reads were then aligned against a ribosomal RNA database using Bowtie2 (v2.2.8); reads failing to map to rRNA were retained for subsequent transcriptomic analysis. The non-ribosomal, paired-end clean reads were aligned to the Mus musculus reference genome (Ensembl release 112) using HISAT2 software (30) (v2.1.0). High-confidence circRNAs were identified by aligning and analyzing the clean reads using CIRI2 software (v2.0.6). Subsequently, the detected circRNA sequences were annotated by aligning them against the circBase database (http://www.circbase.org). This annotation provided essential genomic features, including genomic coordinates, transcript length, exon composition, and source gene information for each circRNA. The transcriptome sequencing data are publicly available under accession number CRA020805 in the Genome Sequence Archive (GSA) database.

### 2.7 RNA sequencing data analysis

Differentially expressed genes between the control groups and the AC model groups were identified using differential expression analysis for sequence count data 2 (DEseq2) software (31). For both mRNA and circRNA datasets, significant differentially expressed genes and circRNAs were screened based on fold change and significance testing results. The criteria for selection included a fold change ≥ 1.5 and a p-value < 0.05. Partial bioinformatic analysis was performed using Omicsmart, a real-time interactive online platform for data analysis (http://www.omicsmart.com).

### 2.8 Functional enrichment analysis

To investigate the mechanisms underlying the differentially expressed circRNAs and mRNAs in AC, we conducted Gene Ontology (GO) and Kyoto Encyclopedia of Genes and Genomes (KEGG) enrichment analyses. These analyses aimed to predict potential signal transduction pathways, biological functions, and biochemical metabolic pathways associated with the differentially expressed RNA. The GO analysis encompasses three ontologies: biological process (BP), cellular component (CC), and molecular function (MF). This framework allows for the annotation of differentially expressed RNAs and facilitates the assessment of their functional significance. Significant enrichment was determined using a hypergeometric test. For Gene Ontology (GO) analysis, a strict threshold of Q-value (corrected P-value) < 0.05 was applied to identify significantly enriched terms. For KEGG pathway analysis, to avoid excluding biologically relevant signaling pathways, a P-value < 0.05 was used as the threshold for significance. Gene Ontology (http://www.geneontology.org/), the KEGG database (http://www.genome.jp/kegg).

### 2.9 Construction of circRNA-mRNA network

To investigate the latent functions of differentially expressed circRNAs and their interactions with mRNAs, we constructed a co-expression network of circRNA–mRNA transcripts. We calculated the Pearson correlation coefficient (PCC) to evaluate the correlation between these circRNAs and mRNAs. The resulting co-expression network, which highlights these significant correlation pairs, was visualized using Cytoscape software (version 3.7.2).

### 2.10 RT-qPCR

The SYBR green RT-qPCR assay was used to confirm the differentially expressed circRNAs and mRNAs identified by transcriptome sequencing. Total RNA isolated from the tissue samples described above was used for reverse transcription and PCR. RT-qPCR was performed using the SYBR green assay kit (TaKaRa Biotechnology, Dalian, China) and a QuantStudio 3 Real-Time PCR System (Thermo Fisher, Waltham, MA, United States). Due to the unique structural characteristics of circRNAs, specific primer design was performed using the specialized software Circprimer 2.0 (32). The specific primers used for RT-qPCR are listed in **Supplementary Table S2**. The RT-qPCR program was as follows: denaturation at 95°C for 30 s, followed by 40 cycles at 95°C for 5s, and 60°C for 34 s. Each sample was run in triplicate. GAPDH was the internal reference of circRNA and mRNA. The relative quantitative analysis of the data was performed by 2^−ΔΔCt^ method. All experiments were performed with at least three biological replicates per group (n=3). To ensure the objectivity of the results, the researchers were blinded to the group assignments during sample processing and data analysis.

### 2.11 Statistical analysis

Statistical analyses for biological experiments (qPCR, flow cytometry, Immune Cell Infiltration analysis, ELISA and histological scoring) were performed using GraphPad Prism 8.0 software (GraphPad Software, San Diego, CA, USA). Data are expressed as the mean ± standard deviation (SD). Differences between the Control and AC model groups, statistical significance was estimated using the unpaired Student’s t-test. A P-value < 0.05 was considered statistically significant.

## 3 Results

### 3.1 Establishment and evaluation of the AC mouse model

**Figure 1:**
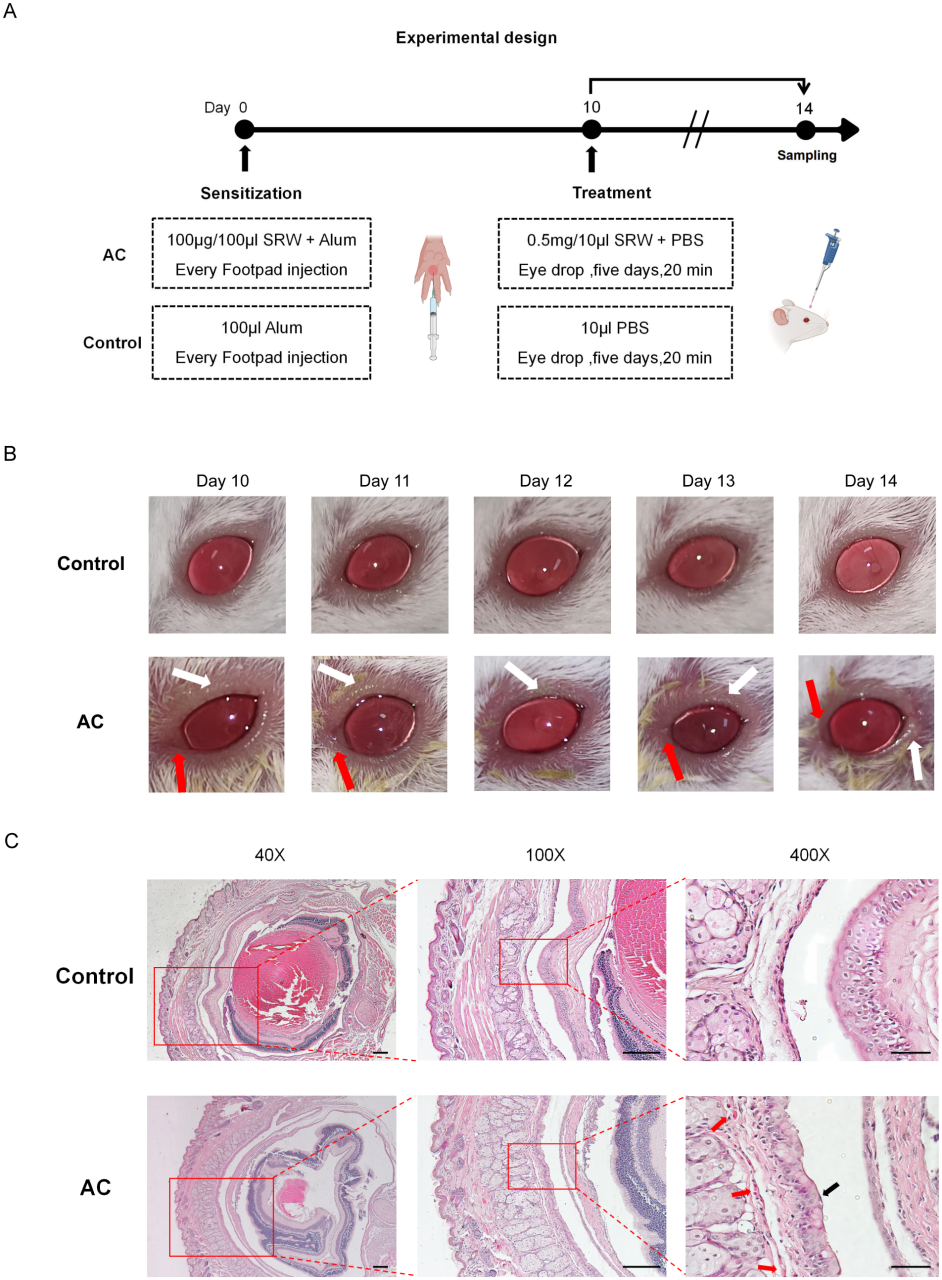
Experimental design and pathological evaluation of the allergic conjunctivitis (AC) model. **(A)** Schematic of the AC modeling procedure. Mice were sensitized on day 0 via footpad injection with 100 μg/100 μl short ragweed pollen (SRW) mixed with alum. From day 10, mice received daily topical ocular administration of 0.5 mg/10 μl SRW in PBS for five consecutive days. **(B)** Representative ocular symptom images in the AC model group (n=5 per group) from day 10 to day 14, showing eyelid edema (white arrows) and mucopurulent discharge (red arrows). **(C)** Representative H&E-stained histopathological sections of mouse eyes. The AC model group (n=5 per group) exhibited massive inflammatory cell infiltration (red arrows) and pathological hyperemic thickening (black arrows). Scale bars: 200 μm (40×), 200 μm (100×), 50 μm (400×).

The allergic conjunctivitis (AC) mouse model was established according to the protocol depicted in **Figure 1A**. Mice were sensitized on day 0 with 100 μg/100 μL of SRW and alum via footpad injection. Starting on day 10, mice received daily eye drops of 0.5 mg/10 μL SRW and PBS for five days. As depicted in **Figure 1B**, the AC model group exhibited pronounced ocular changes compared to the control group, including eyelid edema (white arrows) and bulbar conjunctival swelling (red arrows), which were evident from day 10 to day 14. As shown in **Figure 1C**, H&E staining revealed significant inflammatory cell infiltration in the eyelid conjunctiva (red arrowheads), along with pathological congestion and thickening of the conjunctival tissue (black arrowheads) in the AC model group. These findings confirm the successful establishment of the AC model.

### 3.2 Immune cell infiltration in the AC model

**Figure 2:**
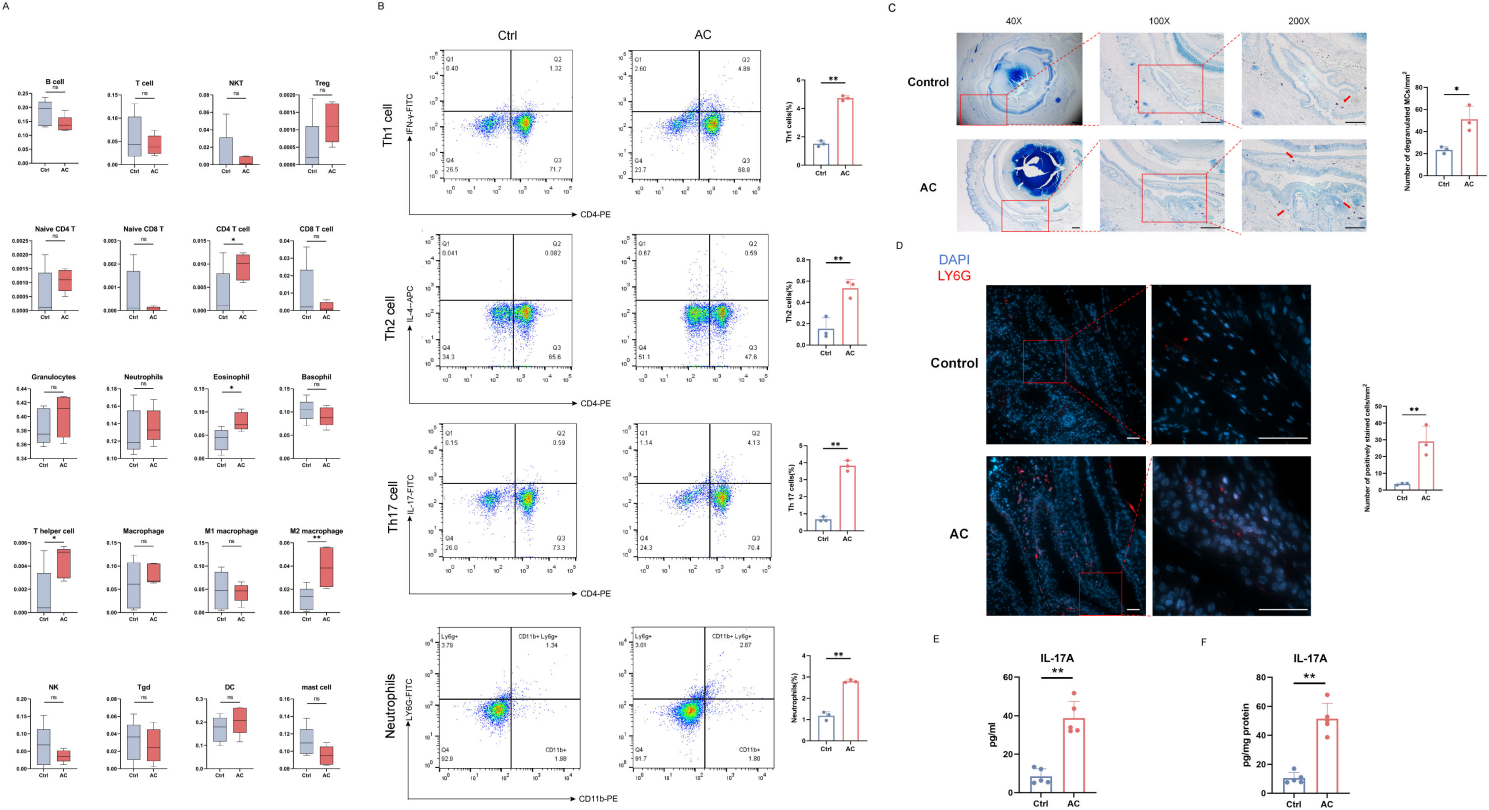
Immune infiltration prediction and multi-platform validation of the Th17/IL-17 axis and neutrophil recruitment. **(A)** Immune infiltration prediction results in the conjunctiva generated using ImmuCellAI-mouse. Statistical significance was determined by Student’s t-test (*P < 0.05, **P < 0.01). **(B)** Representative flow cytometric plots and summarized bar graphs showing the proportions of Th1, Th2, and Th17 cells in cervical lymph nodes (CLNs), and the frequencies of Ly6G^+^ neutrophils in conjunctival single-cell suspensions (n = 3 per group). Statistical significance is indicated by P values (**P < 0.01). **(C)** Representative toluidine blue-stained ocular sections showing mast cell degranulation (red arrows), with quantitative analysis of degranulated mast cells per mm^2^ (n = 3). Scale bars: 200 μm (40×), 200 μm (100×), and 100 μm (200×). **(D)** Representative immunofluorescence images of Ly6G-positive neutrophils (PE-labeled, red) in conjunctival tissues, with DAPI (blue) used for nuclear counterstaining. Quantitative analysis of neutrophils per mm^2^ is shown (n = 3). Scale bars: 50 μm. **(E, F)** Quantitative determination of IL-17A protein levels by ELISA in **(E)** ocular secretion eluates and **(F)** conjunctival tissue homogenates (n = 5 per group). Data are presented as mean ± SD. Statistical significance: *P < 0.05, **P < 0.01.

To characterize the immune cell infiltration profile in the AC model, we utilized transcriptomic sequencing data previously generated from conjunctival tissues of AC model group (33). The relative abundance of various immune cell populations was predicted using the ImmuCellAI-mouse tool based on this transcriptomic data. As shown in **Figure 2A**, the AC model group exhibited a significant increase in the infiltration of CD4^+^ T cells, eosinophils and macrophages in the conjunctival tissues compared to the control group.

To further delineate the immune cell populations involved in the pathogenesis of AC, we performed flow cytometry analysis to assess immune cell dynamics in both lymphoid and mucosal compartments. As shown in **Figure 2B**, the AC model group exhibited a significant increase in the frequencies of Th2 cells within the cervical lymph nodes (CLNs), alongside elevated levels of Th1 and Th17 cell populations. Consistent with this systemic activation, histological analysis confirmed marked mast cell degranulation in the conjunctival tissue (**Figure 2C**). Notably, to evaluate the localized innate immune response, flow cytometric analysis of conjunctival single-cell suspensions revealed a substantial expansion of Ly6G+ neutrophils in the AC model group compared to the control group (**Figure 2B**). This localized recruitment was further corroborated by immunofluorescence staining, which demonstrated a dense infiltration of Ly6G-positive neutrophils (PE-labeled) within the conjunctiva of the AC model group, whereas such cells were rarely observed in the control group (**Figure 2D**).

To determine whether these cellular shifts correlate with functional effector responses at the ocular surface, IL-17A protein levels were quantified via ELISA. The results demonstrated significantly higher levels of IL-17A in both the ocular secretion eluates (**Figure 2E**) and the conjunctival tissue homogenates (**Figure 2F**) of the AC model group relative to the control group. Collectively, these findings suggest that, in addition to the traditional Th2-dominated response orchestrated in the draining lymph nodes, the local activation of the Th17/IL-17 axis and the subsequent recruitment of neutrophils to the conjunctiva play a critical role in the immune dysregulation and pathological progression observed in the AC model group.

### 3.3 Identification and functional analysis of immune-related genes in AC

**Figure 3:**
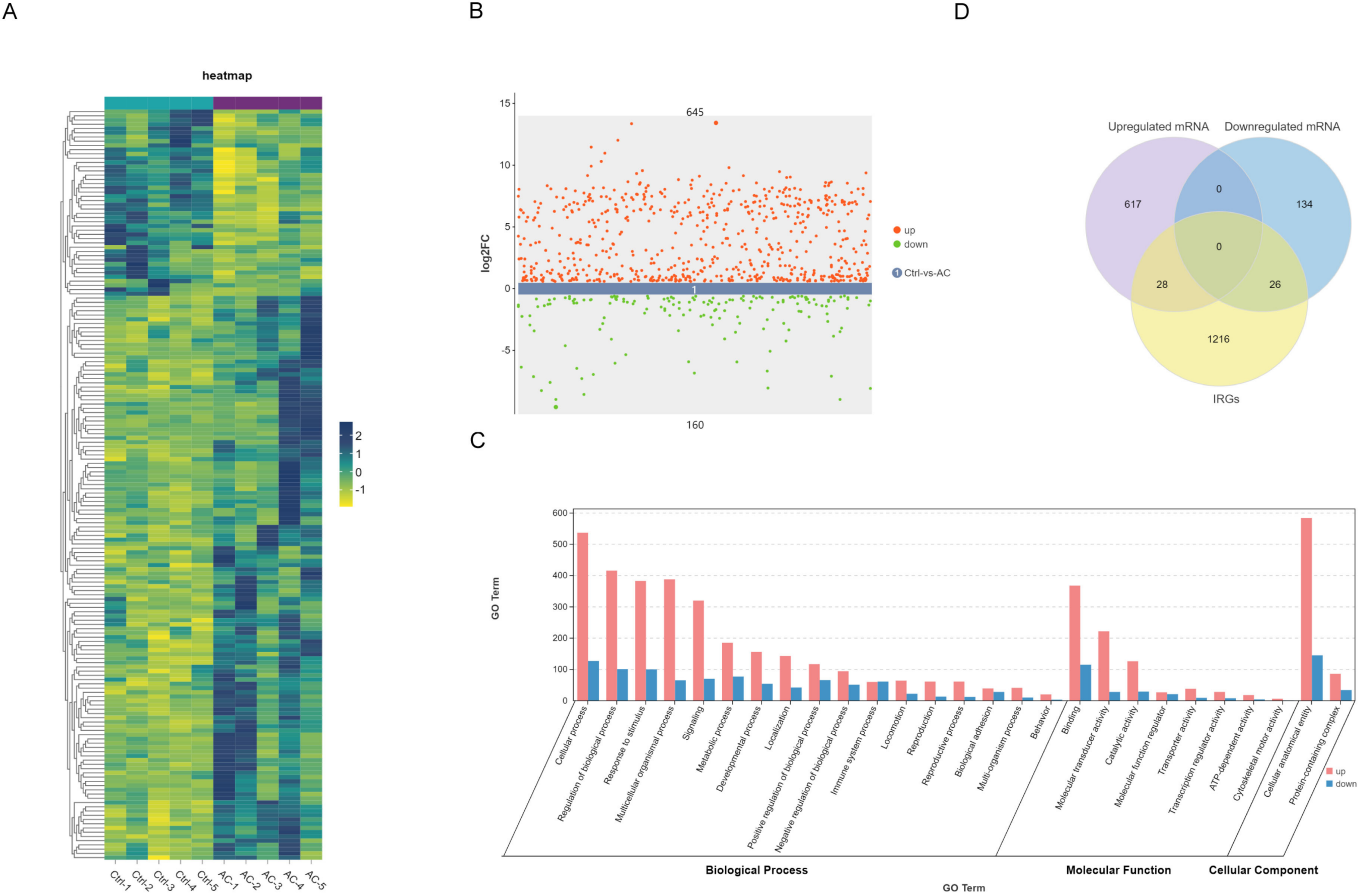
mRNA profiling and immune-related gene (IRG) screening. **(A)** Heatmap depicting differentially expressed mRNA profiles between control and AC samples. **(B)** Volcano plot highlighting significantly upregulated and downregulated genes (|FC| > 1.5, *P*<0.05). **(C)** GO enrichment analysis of upregulated and downregulated differentially expressed genes (DEGs) in AC. **(D)** Venn diagram illustrating overlaps between DEGs and IRGs.

To further explore the immune-related genes potentially involved in the pathogenesis of AC, we analyzed transcriptomic sequencing data from conjunctival tissues of the AC model group (33). Heatmaps illustrate distinct mRNA expression profiles between control and AC samples (**Figure 3A**). A Difference Scatter Plot, generated by applying a fold change (FC) > 1.5 and p-value < 0.05, highlights 645 upregulated genes and 160 downregulated genes in the AC samples (**Figure 3B**).

To investigate the biological functions of these differentially expressed genes, GO enrichment analysis was performed. As shown in **Figure 3C**, upregulated genes were significantly enriched in biological processes related to response to stimulus, multicellular organismal processes, signaling, metabolic processes, developmental processes, and immune system processes. In terms of molecular function, binding activity was most prominently represented, with additional enrichment in catalytic activity and molecular transducer activity. In the cellular component category, cellular anatomical entities and protein-containing complexes were notably enriched. Similarly, downregulated genes also exhibited some enrichment in these pathways.

Given the critical involvement of the immune system in AC, we further focused on identifying immune-related genes. As shown in **Figure 3D**, a Venn diagram illustrates the overlap between differentially expressed mRNAs and IRGs, revealing 28 upregulated and 26 downregulated IRGs.

### 3.4 GO and KEGG analysis of immune-related genes in AC

**Figure 4:**
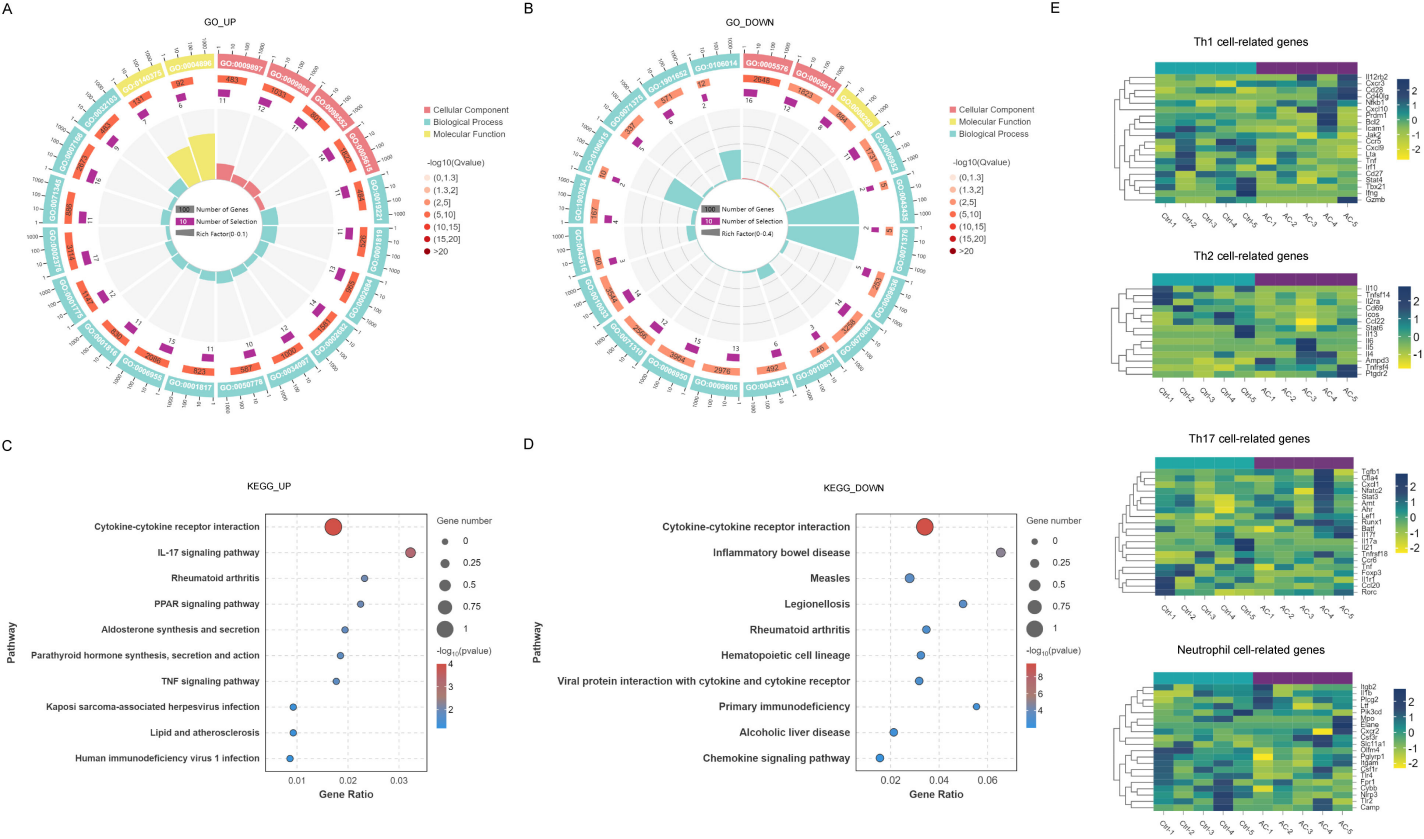
GO and KEGG enrichment analyses of differentially expressed IRGs in AC. GO enrichment of upregulated **(A)** and downregulated **(B)** IRGs across biological processes, molecular functions, and cellular components. Color intensity reflects statistical significance expressed as -log_10_(Q-value). KEGG pathway enrichment of upregulated **(C)** and downregulated **(D)** IRGs. Dot size indicates gene count per pathway; color intensity reflects statistical significance (-log_10_(*P*-value)). **(E)** Heatmap of Th1/Th2/Th17-related and neutrophil-associated mRNA expression profiles.

To further investigate the inflammation-related signaling pathways implicated in AC, we conducted GO enrichment analysis of the upregulated IRGs. The analysis revealed significant enrichment in various biological processes, molecular functions, and cellular components. Upregulated IRGs were notably involved in biological processes such as cytokine-mediated signaling (GO:0019221), positive regulation of cytokine production (GO:0001819), positive regulation of response to external stimuli (GO:0032103), positive regulation of immune response (GO:0050778), and cytokine production (GO:0001816). In terms of molecular function, immune receptor activity (GO:0140375) and cytokine receptor activity (GO:0004896) were prominently represented. Regarding cellular components, the external side of the plasma membrane (GO:0009897) was significantly enriched (**Figure 4A**).

Conversely, downregulated IRGs were primarily associated with processes such as response to corticotropin-releasing hormone (GO:0043435) and cellular response to corticotropin-releasing hormone stimulus (GO:0071376), as well as the negative regulation of the inflammatory response to wounding (GO:0106015). In terms of molecular functions, lipid binding (GO:0008289) was notably enriched, along with cellular components such as the extracellular region (GO:0005576) and extracellular space (GO:0005615) (**Figure 4B**).

KEGG pathway analysis of the upregulated IRGs (**Figure 4C**) revealed their involvement in pathways such as cytokine-cytokine receptor interaction, IL-17 signaling, PPAR signaling, and TNF signaling. For downregulated IRGs, the analysis highlighted significant involvement in the cytokine-cytokine receptor interaction and chemokine signaling pathways (**Figure 4D**). **Figure 4E** presents heatmaps illustrating the gene expression profiles related to Th1, Th2, Th17 cells, and neutrophils in the AC model group, further elucidating the immune cell subsets involved in the inflammatory processes.

### 3.5 Characterization of circRNA expression profiles

**Figure 5:**
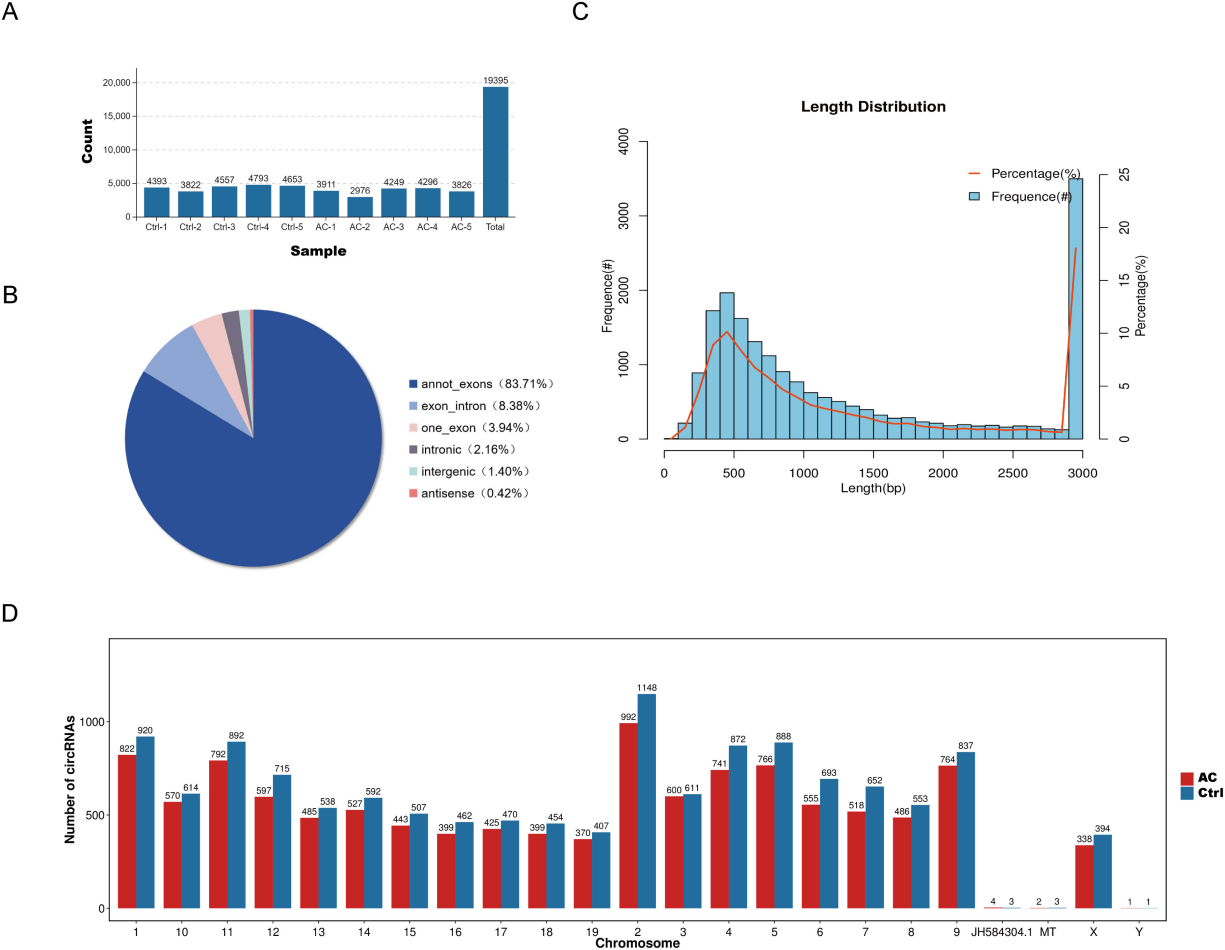
Characterization of circRNAs. **(A)** CircRNA distribution across samples. **(B)** Classification of circRNAs types. **(C)** Expression characteristics by circRNA length distribution. **(D)** Chromosomal distribution of circRNAs per sample.

CircRNAs, a class of non-coding RNAs, are involved in various biological processes, but their roles in the pathogenesis of AC remain unclear. To explore their potential involvement in AC, we analyzed the expression profiles of circRNAs across all samples. A total of 19,395 circRNAs were identified (**Figure 5A**), with lengths ranging from 80 to over 3,000 nucleotides, with the majority around 3,000 nucleotides (**Figure 5C**). Based on their genomic origins, circRNAs were classified into six categories: annot_exons (83.71%, n = 16,231), exon_intron (8.38%, n = 1,625), one_exon (3.94%, n = 763), intronic (2.16%, n = 419), intergenic (1.40%, n = 271), and antisense (0.42%, n = 81) (**Figure 5B**). Chromosomal distribution analysis showed that the control group had slightly more circRNAs than the experimental group (**Figure 5D**).

### 3.6 Functional analysis of differentially expressed circRNAs in AC

**Figure 6:**
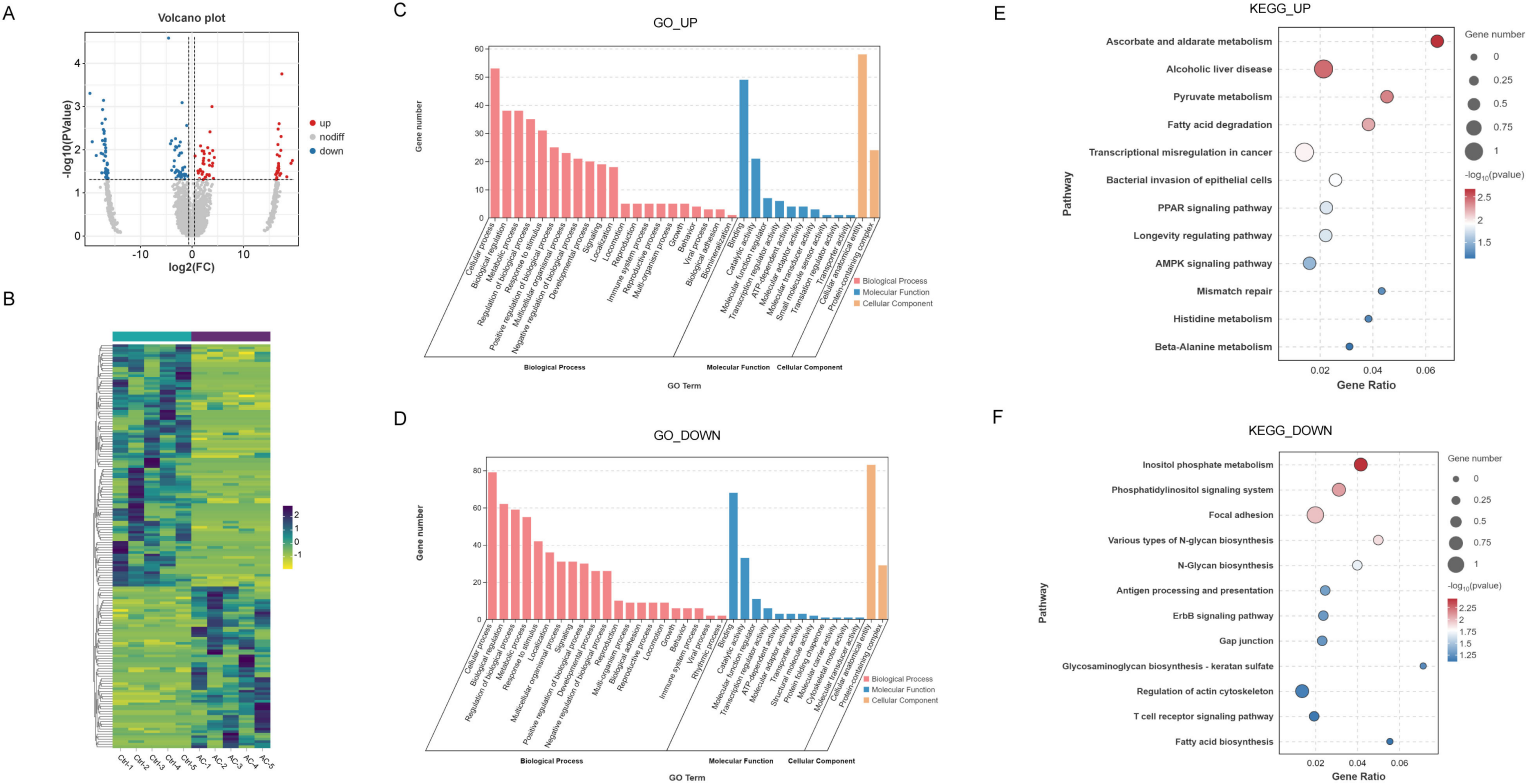
CircRNA analysis. **(A)** Volcano plot of differentially expressed circRNAs (|FC| > 1.5, *P*<0.05). **(B)** Heatmap of circRNA expression profiles. GO enrichment of host genes for upregulated **(C)** and downregulated **(D)** circRNAs. KEGG enrichment of host genes for upregulated **(E)** and downregulated **(F)** circRNAs. Dot size indicates gene count; color intensity reflects -log_10_(*P*-value).

To investigate the expression changes of circRNAs in AC, we analyzed the expression profiles of circRNAs based on high-throughput sequencing data. First, we normalized the expression levels of all circRNAs across samples and generated a clustering heatmap (**Figure 6A**). The results revealed significant differences in overall expression patterns between AC and control samples. Subsequently, differentially expressed circRNAs were screened using differential expression analysis. As shown in **Figure 6B**, circRNAs meeting the threshold of FC > 1.5 and p-value < 0.05 were identified as statistically significant differentially expressed circRNAs. Among these, 64 circRNAs were upregulated and 96 were downregulated.

To explore the biological functions of these differentially expressed circRNAs, GO enrichment analysis and KEGG pathway analysis were performed on the source genes of these circRNAs. GO enrichment analysis of the source genes of upregulated circRNAs revealed significant enrichment in biological processes such as cellular process, biological regulation, metabolic processes, response to stimulus, positive regulation of biological process and immune system process. In terms of molecular function, binding, catalytic activity and molecular transducer activity were prominently enriched, and cellular anatomical entities and protein-containing complexes were significantly enriched within cellular components (**Figure 6C**). GO enrichment analysis of source genes of downregulated circRNAs revealed that their functional annotations also predominantly clustered around classical core biological processes. In biological process, enrichment was primarily associated with cellular process, metabolic processes and biological regulation. In molecular functions, binding and catalytic activity were significantly enriched; In cellular components, cellular anatomical entities and protein-containing complexes were also enriched (**Figure 6D**).

To further explore the potential molecular mechanisms of differentially expressed circRNAs in the development of AC, we performed KEGG pathway enrichment analysis on the source genes of both upregulated and downregulated circRNAs. The results, shown in **Figure 6E**, indicate that the source genes of upregulated circRNAs were significantly enriched in multiple classical signaling pathways associated with metabolic regulation and inflammation, including Ascorbate and aldarate metabolism (ko00053), Bacterial invasion of epithelial cells (ko05100), PPAR signaling pathway (ko03320), and AMPK signaling pathway (ko04152). These findings suggest that upregulated circRNAs may play a crucial role in the pathogenesis of AC by regulating energy metabolism, inflammatory responses, and epithelial barrier function. In contrast, the source genes of downregulated circRNAs were primarily enriched in multiple pathways related to immune regulation, signal transduction, and glycosylation (**Figure 6F**), including Inositol phosphate metabolism (ko00562), Phosphatidylinositol signaling system (ko04070), T cell receptor signaling pathway (ko04660) and Antigen processing and presentation (ko04612). This suggests that downregulated circRNAs may participate in inflammatory immune regulation in AC by influencing immune responses, cell recognition, and signaling processes.

### 3.7 Identification and validation of IL-17 signaling pathway-upregulated genes and their co-expressed circRNAs in AC

**Figure 7:**
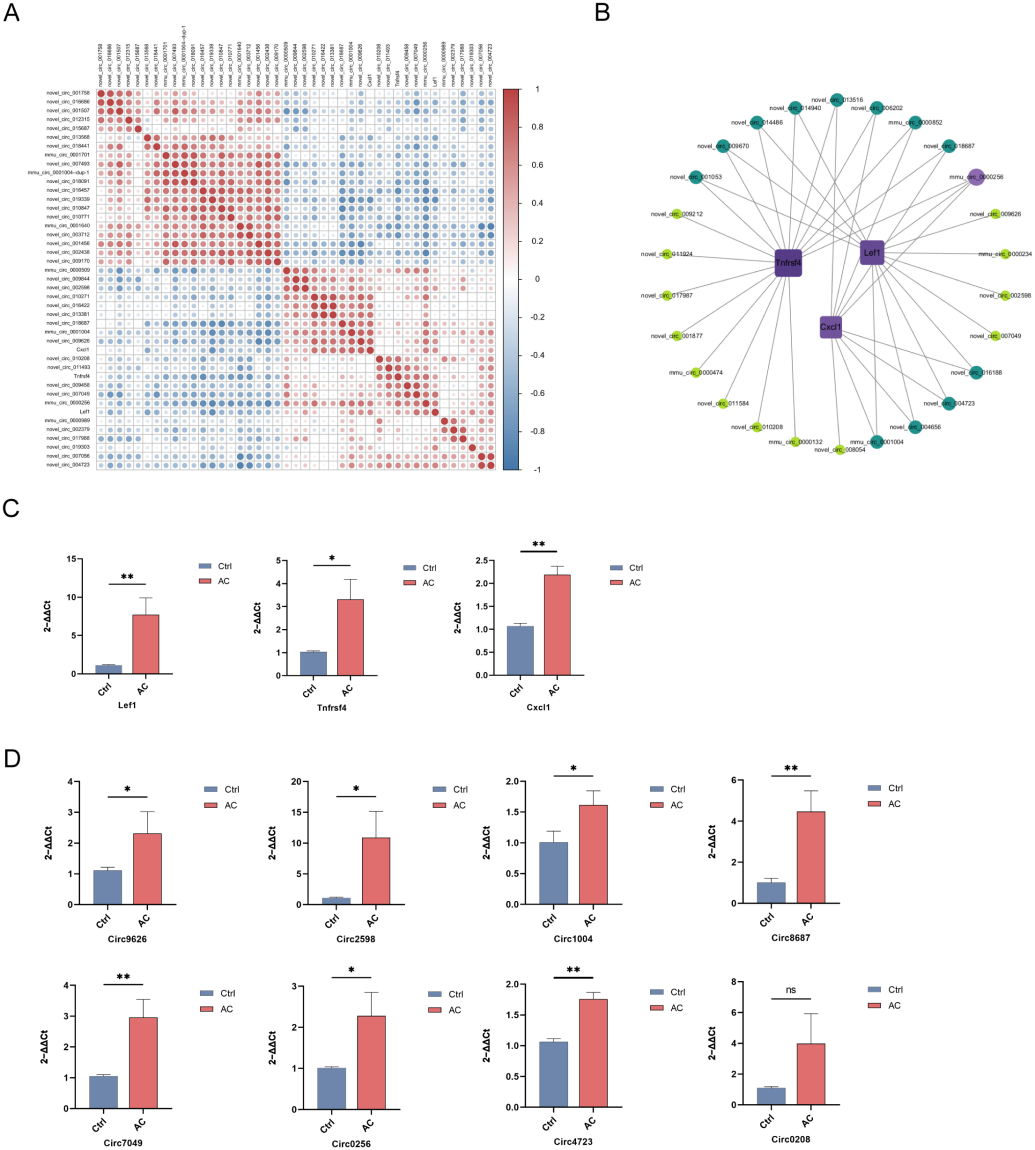
Construction and validation of the co-expression network between key IL-17 pathway genes and circRNAs **(A)** Correlation heatmap between 3 key IL-17 pathway mRNAs (*Cxcl1*, *Tnfrsf4*, *Lef1*) and top 40 upregulated circRNAs. Blue/red indicate negative/positive correlations; color intensity reflects correlation strength. **(B)** Co-expression network of 3 key IL-17 pathway mRNAs and positively correlated circRNAs. Node size represents correlation coefficient; node color indicates number of co-expressed genes (darker = more connections). qPCR validation of mRNA **(C)** and top 8 correlated circRNAs **(D)** expression in conjunctival tissues of control and AC model group. All data (mean ± SD) were obtained from 3 mice per group. Statistical significance was determined by Student’s t-test (*P<0.05, **P<0.01).

In our preliminary studies, we observed that, in addition to the traditional involvement of Th2 cells in AC, Th17 cells also play a significant role in its pathogenesis. As depicted in **Figure 4B**, the IL-17 signaling pathway was markedly upregulated in the AC model. To further explore this finding, three genes linked to IL-17 signaling, *Tnfrsf4*, *Cxcl1*, and *Lef1*, were chosen as candidates to analyze their expression profiles in AC. To identify potential circRNAs involved in regulating the IL-17 signaling pathway, we performed Pearson correlation analysis to examine the relationship between the expression levels of these IL-17 pathway genes and differentially expressed circRNAs, as depicted in **Figure 7A**. A co-expression network was then constructed to visualize the circRNAs that were positively correlated with IL-17 pathway-related genes (**Figure 7B**).

To validate these findings, we performed qPCR to assess the expression of the selected genes and their co-expressed circRNAs in the AC model group. As shown in **Figure 7C**, the expression levels of *Tnfrsf4*, *Cxcl1*, and *Lef1* were significantly upregulated in the AC model group compared to the control group. Additionally, several circRNAs, including Circ9626, Circ2598, Circ1004, Circ8687, Circ7049, Circ0256, and Circ4723, were notably elevated in the AC model group (**Figure 7D**). These results suggest that these genes and circRNAs may contribute to the increased IL-17 signaling observed in AC.

## 4 Discussion

Allergic conjunctivitis (AC) represents a prevalent ocular surface inflammatory condition, conventionally categorized as a type I hypersensitivity reaction predominantly orchestrated by Th2 cells. The Th2-derived cytokines, including IL-4, IL-5, and IL-13, play pivotal roles in IgE production, mast cell activation, and eosinophil recruitment, thereby driving the pathogenesis of conjunctival inflammation (34). However, ocular immunity, as a specialized component of mucosal immunity, exhibits unique regulatory mechanisms to maintain ocular homeostasis and prevent excessive inflammatory damage. Despite extensive research focusing on the classical Th2-mediated response in AC, the distinctive immune regulatory pathways in the ocular microenvironment remain poorly understood (35). In this study, using a ragweed pollen-induced murine AC model integrated with transcriptomic sequencing, we systematically investigated the immunoregulatory mechanisms of AC, with particular emphasis on the activation of the Th17/IL-17 signaling pathway and its potential interplay with the circRNAs regulatory network. These findings provide new insights into the complex immunological mechanisms underlying AC and highlight potential therapeutic targets for this condition.

AC is primarily mediated by Th2 cells, and our findings align with this established mechanism. Histological analysis using H&E staining revealed increased infiltration of inflammatory cells in the conjunctival tissue, and flow cytometry confirmed an elevated proportion of Th2 cells. Additionally, toluidine blue staining demonstrated enhanced mast cell degranulation in the conjunctiva, consistent with the characteristic allergic response. However, recent studies suggest that the immunological mechanisms of AC may extend beyond the classical Th2-driven response. While Th2 cells and their associated cytokines dominate the acute phase of allergic inflammation, emerging evidence indicates the involvement of additional immune pathways in disease progression. For example, Th1 responses, characterized by IFN-γ production, have been observed in certain subtypes of AC, suggesting a potential modulatory role in inflammation (36). Furthermore, regulatory T cells (Tregs), through the secretion of IL-10 and TGF-β, may exert suppressive effects that partially counteract conjunctival inflammation and contribute to immune homeostasis.

In the present study, our data demonstrate a distinct activation of Th17-related pathways paralleling the development of AC. However, dissecting whether the Th17 response arises independently, operates synergistically with Th2 immunity, or occurs as a secondary consequence of Th2-driven inflammation remains a critical challenge. Emerging immunological evidence suggests that, particularly in severe allergic pathologies, Th2 and Th17 responses are not merely antagonistic but may instead exhibit complex synergistic regulation (37). On one hand, IL-17 may directly amplify Th2-mediated inflammatory signaling. For instance, IL-17A has been shown to exacerbate allergic responses by enhancing IL-13-induced STAT6 phosphorylation; conversely, IL-17 receptor-deficient mice exhibit attenuated Th2 responses and impaired germinal center formation (38). On the other hand, cellular plasticity represents a pivotal driver of mixed-type inflammation. In asthma models, a “pathogenic Th2/Th17 hybrid subset” (or IL-17-producing Th2 cells) co-expressing GATA-3 and RORγt has been identified (37, 39). These cells possess the dual capacity to recruit both eosinophils and neutrophils, frequently resulting in more severe pathological damage than that elicited by a singular Th2 response (37, 39). This mechanism aligns closely with the mixed granulocytic infiltration phenotype observed in our AC model, suggesting that Th17 signaling may exacerbate ocular surface injury by orchestrating inflammatory cell recruitment and sustaining chronic inflammatory loops, such as the IL-23/Th17 axis (38). Nevertheless, definitively dissecting the precise role of Th17 in AC progression requires in-depth verification via lineage tracing or dual gene knockout strategies.

In this study, immune cell infiltration in the conjunctival tissue was predicted using ImmuCellAI-mouse, which identified significant infiltration of CD4^+^ T cells, eosinophils, and macrophages. Flow cytometry further revealed an increase in Th1 and Th17 cells in the AC model group, with particular emphasis on the role of Th17 cells and their effector neutrophils. These findings suggest a potential involvement of Th17-mediated type III immunity in the pathogenesis of AC.

Th17 cells have garnered increasing attention in the field of ocular allergies because of their strong pro-inflammatory characteristics (40). Defined by the production of IL-17A, IL-17F, IL-21, and IL-22, Th17 cells promote neutrophil chemotaxis and activation while enhancing the expression of pro-inflammatory mediators such as IL-6 and CXCL1. Previous studies have shown that Th17 cells, through the production of IL-17A and IL-22, contribute to ocular surface epithelial apoptosis and lymphangiogenesis, thereby facilitating immune cell trafficking to draining lymphoid tissues (41). These findings highlight the role of Th17-mediated responses in amplifying conjunctival inflammation in AC.

IL-17, a key effector molecule of Th17 cells, plays a significant pro-inflammatory role. It induces the expression of chemokines and adhesion molecules, promoting neutrophil recruitment and exacerbating local inflammatory responses in various tissues. In ocular diseases, activation of IL-17-related signaling pathways has been associated with the pathological progression of conditions such as dry eye and uveitis (42). Notably, a recent study demonstrated that IL-17 plays a pivotal role in particulate matter-induced AC and allergic rhinitis, where IL-17 neutralization reduced ocular and nasal inflammation and decreased neutrophil infiltration (43).

Transcriptomic analysis revealed a significant upregulation of the IL-17 signaling pathway in the conjunctival tissue of AC model group. Key genes within this pathway, including *Tnfrsf4*, *Cxcl1*, and *Lef1*, exhibited marked elevation, suggesting a pivotal role of the Th17-IL-17 axis in the pathogenesis of AC. *Tnfrsf4*, also known as OX40, is a T cell activation-associated receptor. It enhances the sustained activation of Th17 cells, amplifying their pro-inflammatory effects (44). *Cxcl1*, a classical neutrophil chemokine, directly mediates the infiltration of inflammatory cells, contributing to tissue inflammation (45). *Lef1*, a critical transcription factor in the Wnt signaling pathway, is closely associated with Th17 cell differentiation and function (46). Consistent with these transcriptomic signatures, our ELISA analysis confirmed a significant elevation of IL-17A protein levels in both ocular secretions and conjunctival tissues. Furthermore, immunofluorescence staining identified a substantial influx of Ly6G-positive neutrophils within the conjunctival tissue of the AC model group. These findings collectively underscore the potential involvement of the Th17-IL-17 axis in driving the inflammatory cascade in AC. Notably, as these results reflect a composite signal from bulk tissue sequencing, future investigations with single-cell resolution are warranted to precisely delineate the specific cellular sources.

CircRNAs, a class of stable, non-coding RNAs with regulatory potential, are increasingly recognized for their roles in immune modulation and inflammation (47, 48). However, their relevance in AC has not been previously explored. To investigate the potential role of circRNAs in AC, we performed circRNA sequencing analysis on the conjunctival tissue of AC model group and identified several significantly differentially expressed circRNAs. GO functional annotation revealed that the source genes of these circRNAs were predominantly enriched in core biological processes such as cellular processes, biological regulation, and metabolic processes. At the molecular functional level, the circRNAs were primarily involved in binding activity and catalytic activity. KEGG pathway analysis further revealed that the upregulated circRNA-associated genes were enriched in inflammation regulation, epithelial cell function, and metabolism-related pathways, including the PPAR signaling pathway, AMPK signaling pathway, and epithelial cell invasion. In contrast, the downregulated circRNA-associated genes were enriched in immune regulation pathways, including T cell receptor signaling, antigen processing and presentation, and phospholipid signaling. These findings suggest that circRNAs may participate in the pathogenesis of AC by influencing immune responses, energy metabolism, and epithelial barrier functions.

To explore the relationship between circRNAs and the IL-17 signaling pathway, we further identified circRNAs that were significantly positively correlated with the expression levels of *Tnfrsf4*, *Cxcl1*, and *Lef1*. Co-expression network analysis revealed that several circRNAs, including Circ9626, Circ2598, Circ1004, Circ8687, Circ7049, Circ0256, and Circ4723, were significantly correlated with IL-17 pathway-related genes, indicating that they may play a role in modulating the expression of these key molecules in AC-related inflammation. To validate these findings, we performed RT-qPCR to measure the expression levels of the upregulated IL-17 signaling pathway genes (*Tnfrsf4*, *Cxcl1*, and *Lef1*) and eight associated circRNAs. The results demonstrated that, compared to the control group, the expression levels of *Tnfrsf4*, *Cxcl1*, and *Lef1* were significantly elevated in the AC model group. Furthermore, seven of the eight selected circRNAs (Circ9626, Circ2598, Circ1004, Circ8687, Circ7049, Circ0256, and Circ4723) exhibited a significant upregulation trend in the AC model group. These results indicate a strong statistical association, suggesting that these circRNAs may serve as potential upstream modulators in the IL-17 signaling network.

Our previous investigation revealed a significant upregulation of several antimicrobial peptide-related genes in the conjunctiva, indicating an enhanced mucosal antimicrobial capacity following AC (33). Th17 cells, primarily through the secretion of IL-17, play a pivotal role in antimicrobial immunity. IL-17 not only stimulates the production of antimicrobial peptides but also promotes the recruitment of neutrophils to sites of infection, thereby bolstering the host’s defense against microbial invasion (40, 49). Given that the conjunctival epithelium is continuously exposed to environmental pathogens and allergens, observed induction of antimicrobial peptide-related genes, coupled with an enhanced Th17-mediated immune response, may represent a specialized defense mechanism of the conjunctiva during allergic inflammation. Although AC-induced inflammation compromises the integrity of the ocular mucosal barrier, the concurrent augmentation of antimicrobial immune responses could serve as a compensatory mechanism to reduce the risk of secondary microbial infections.

Notably, the mechanisms underlying the enhancement of mucosal antimicrobial immunity following AC remain incompletely understood. Although some studies have reported alterations in the ocular surface microbiota during AC, infections are not a typical direct complication of this condition, and our observations in mice did not reveal significant signs of ocular infection (50–52). Therefore, the upregulation of antimicrobial peptides (AMPs) and Th17 cells may not be solely driven by microbial infection but rather represents a pre-adaptive response of the conjunctiva to allergen exposure, aimed at enhancing local immune defense capabilities. Despite these insights, the mechanisms responsible for the upregulation of these molecules during AC induction remain unclear. Furthermore, the roles of these molecules in influencing the progression and severity of AC are yet to be fully elucidated. These questions warrant further investigation to clarify the complex interplay between antimicrobial immunity and allergic inflammation.

Building upon this perspective of mucosal defense, our observation of a robust Th17/IL-17 signature in the AC model is further corroborated by clinical evidence. Yan et al. reported significantly elevated IL-17 levels in the tears of children with AC, which positively correlated with disease severity markers, highlighting its clinical relevance in ocular allergies (53). Our findings mirror these clinical observations, demonstrating that the upregulation of IL-17A and the subsequent recruitment of neutrophils as visualized by Ly6G staining are functional components of the allergic ocular environment rather than mere transcriptomic byproducts. However, the functional role of IL-17 on the ocular surface appears multifaceted and context-dependent. Beyond its pro-inflammatory potential, IL-17 serves as a critical component of homeostatic barrier defense. Specifically, St. Leger et al. demonstrated through functional models that IL-17, primarily derived from γδ T cells, is essential for protecting the cornea against pathogenic infections (53). This underscores a sophisticated interplay between the lacrimal cytokine milieu, conjunctival intraepithelial lymphocytes, and the ocular microbiota, as further discussed by Zarzuela et al. (54). Consequently, the concurrent upregulation of the IL-17 pathway and antimicrobial peptides observed in our study may represent a compensatory or pre-adaptive mechanism aimed at reinforcing mucosal integrity during allergic insult. While our data establish a strong transcriptomic association, future studies employing IL-17 neutralization or lineage-specific knockouts are required to definitively determine whether this pathway serves as a primary driver of conjunctival injury or a protective feedback response.

In conclusion, AC is a common ocular surface inflammatory disorder traditionally attributed to Th2-driven type I hypersensitivity. Using a ragweed-induced murine AC model combined with transcriptomic profiling, we demonstrated that, in addition to classical Th2 activation, Th17/IL-17 signaling contributes to the pathogenesis, with key genes such as *Tnfrsf4*, *Cxcl1*, and *Lef1* significantly upregulated. Furthermore, specific circRNAs were identified as potential candidates significantly correlated with IL-17 pathway activation. These findings provide a comprehensive transcriptomic landscape of AC and offer promising targets for future mechanistic validation and therapeutic exploration.

## Data Availability

The data presented in the study are deposited in the Genome Sequence Archive (GSA) repository, accession number CRA020805 (associated with BioProject PRJCA031566): https://ngdc.cncb.ac.cn/gsa/browse/CRA020805.
